# Repurposing ebselen for treatment of multidrug-resistant staphylococcal infections

**DOI:** 10.1038/srep11596

**Published:** 2015-06-26

**Authors:** Shankar Thangamani, Waleed Younis, Mohamed N. Seleem

**Affiliations:** 1 Department of Comparative Pathobiology, College of Veterinary Medicine, Purdue University, West Lafayette, Indiana, USA

## Abstract

Novel antimicrobials and new approaches to developing them are urgently needed. Repurposing already-approved drugs with well-characterized toxicology and pharmacology is a novel way to reduce the time, cost, and risk associated with antibiotic innovation. Ebselen, an organoselenium compound, is known to be clinically safe and has a well-known pharmacology profile. It has shown potent bactericidal activity against multidrug-resistant clinical isolates of *staphylococcus aureus*, including methicillin- and vancomycin-resistant *S. aureus* (MRSA and VRSA). We demonstrated that ebselen acts through inhibition of protein synthesis and subsequently inhibited toxin production in MRSA. Additionally, ebselen was remarkably active and significantly reduced established staphylococcal biofilms. The therapeutic efficacy of ebselen was evaluated in a mouse model of staphylococcal skin infections. Ebselen 1% and 2% significantly reduced the bacterial load and the levels of the pro-inflammatory cytokines tumor necrosis factor-α (TNF-α), interleukin-6 (IL-6), interleukin-1 beta (IL-1β), and monocyte chemo attractant protein-1 (MCP-1) in MRSA USA300 skin lesions. Furthermore, it acts synergistically with traditional antimicrobials. This study provides evidence that ebselen has great potential for topical treatment of MRSA skin infections and lays the foundation for further analysis and development of ebselen as a potential treatment for multidrug-resistant staphylococcal infections.

In 2013, the Centers for Disease Control and Prevention (CDC) reported that more than 11,000 people died from a methicillin-resistant *Staphylococcus aureus* (MRSA)-related infection in the United States; this figure represents nearly half of all fatalities caused by antibiotic-resistant bacteria. Apart from the high mortality rate, *S. aureus* is the most common pathogen associated with skin and soft tissue infections in humans^1^,^2^,^3^,^4^ . Furthermore, *S. aureus* and its secreted toxins, and ability to form biofilm, are responsible for interfering with the wound-healing process and causing systemic complications in affected patients. In addition, the rising prevalence of multidrug-resistant *S. aureus* strains and the extensive use of drugs of choice increase the likelihood that more challenging–to-treat isolates will become a new scourge. Without a doubt, novel antimicrobials and novel approaches to developing them are urgently needed; however, new antimicrobials are becoming increasingly difficult to develop and are currently unable to keep pace with the emergence of resistant bacteria^5^. The concept of repurposing drugs to find new applications outside the scope of their original medical indication is recently gaining much attention and has resulted in successes in a number of disease areas^6^,^7^. Unlike *de novo* drug discovery, repurposing old drugs with known pharmacology and toxicology greatly reduces the time, cost, and risk associated with antibiotic innovation^7^,^8^,^9^. In an attempt to repurpose non-antibiotic drugs as antimicrobial agents, we screened National Institute of Health (NIH) Clinical Collection library against MRSA^6^. Ebselen (2-phenyl-1, 2-benzisoselenazol-3(2*H*)-one, PZ51), a selenium-containing compound, showed potent activity, in an applicable clinical range, against *S. aureus,* which is in agreement with the previous finding^10^.

Previous studies reported that ebselen possesses anti- atherosclerotic, anti-inflammatory and antioxidative properties^11^,^12^,^13^,^14^. In addition, antimicrobial properties of ebselen has also been explored. It has been shown to inhibit yeast and *Escherichia coli in vitro*^10^,^15^. It interferes with proton-translocation function and ATPase activity in yeast, while in *E. coli,* it inhibits the thioredoxin reducatse (TrxR) enzyme^15^,^16^. However, clinical applications and the underlying mechanism of action for its antibacterial activity against *S. aureus* still remain unclear^15^.

Thus, the aim of our study is to assess the antibacterial action of ebselen and its spectrum of activity against clinical isolates of MRSA; to investigate its antimicrobial mechanism of action, anti-biofilm activity, and effect on toxin production in MRSA; and finally to validate its antimicrobial efficacy, anti-inflammatory properties, and potential clinical applications in MRSA infected animal model.

## Results

### Antibacterial activity of ebselen

The antimicrobial activity of ebselen was tested against a panel of clinical isolates of multi-drug resistant *S. aureus* (Table 1). Ebselen showed potent bactericidal activity against MRSA, vancomycin-resistant *S. aureus* (VRSA), linezolid-resistant *S. aureus*, mupirocin-resistant *S. aureus,* methicillin-resistant *S. epidermidis*, and multidrug-resistant strains with a minimum inhibitory concentration (MIC) ranging from 0.125 μg/ml to 0. 5 μg/ml (Table 2).

### Mechanism of action

**Figure 1:**
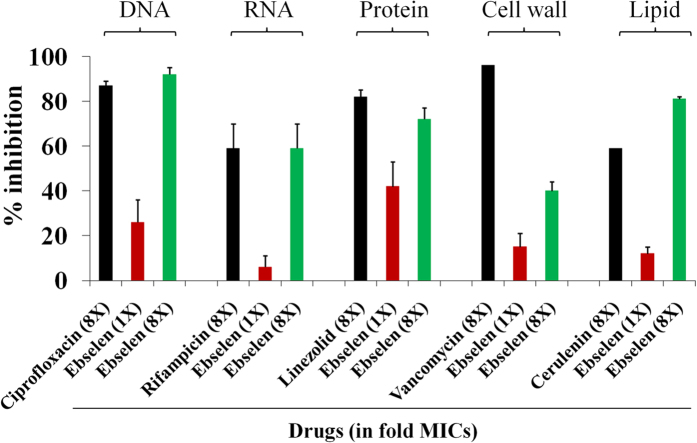
Macromolecular synthesis in the presence of ebselen. Incorporation of radiolabeled precursors of DNA, RNA, protein, cell wall and lipid synthesis ([3H] thymidine, [3H] uridine, [3H] leucine, [14C] N-acetylglucosamine and [3H] glycerol, respectively) were quantified in *S. aureus* ATCC 29213. Results were expressed as percent of inhibition calculated based on the incorporation of radiolabeled precursors.

Given the potent anti-staphylococcal activity of ebselen *in vitro*, we investigated its anti-staphylococcal mechanism of action by macromolecular synthesis assay. As shown in Fig. 1, ebselen primarily inhibited protein synthesis at 1X the MIC. However, additional secondary effects were observed at a higher concentration (8X MIC). At higher concentration, ebselen inhibited DNA, RNA and lipid synthesis similar to control antibiotics such as ciprofloxacin, rifampicin and cerulenin respectively.

### Ebselen inhibits MRSA toxin production

**Figure 2:**
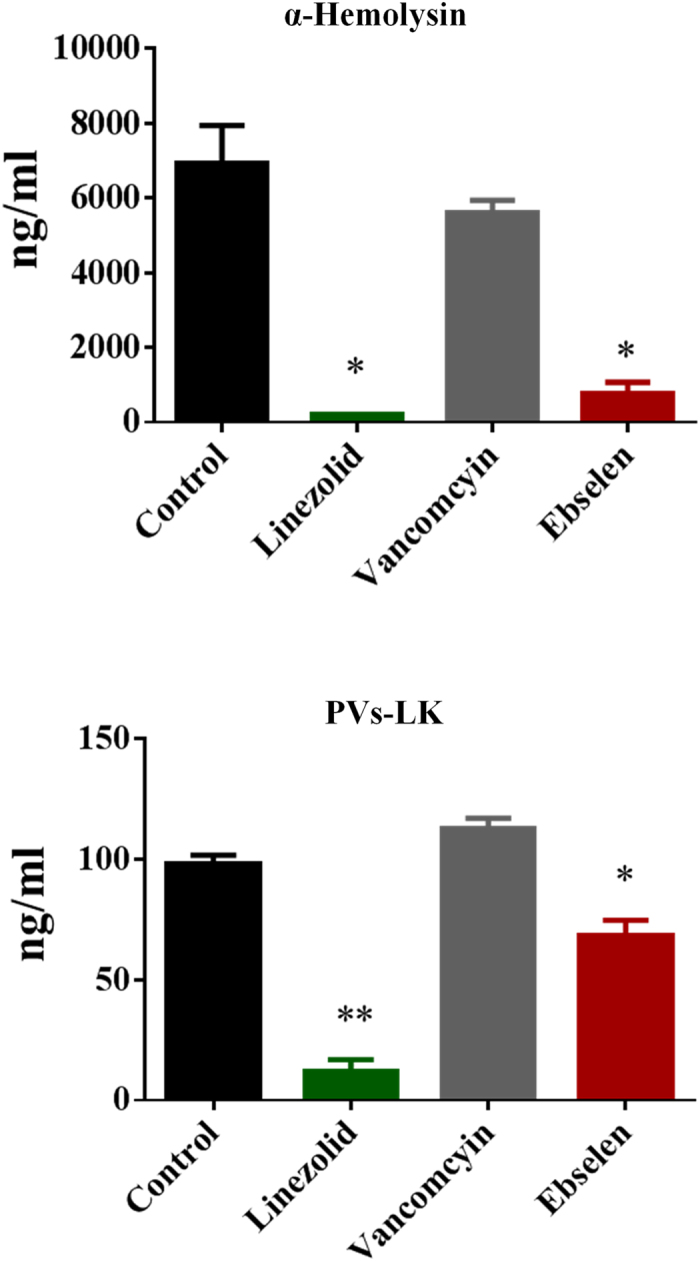
Effect of ebselen on toxin production. Toxin production (ng/ml) in *S. aureus* MRSA USA300 after antibiotic/drug exposure for 1 hour corrected for organism burden. The results are given as means ± SD (n = 3). **indicate statistical significant different from control (DMSO or water). *P* values of (*P ≤ 0.05) (**P ≤ 0.01) are considered as significant. Detailed *P* values are listed: α Hemolysin: Control vs linezolid, 0.0144; Control vs ebselen, 0.0147. PVs-LK: Control vs linezolid, 0.0024; Control vs ebselen, 0.0288.

The effect of ebselen on production of important toxins such as Panton-Valentine leucocidin (PVL) and α-hemolysin (Hla) was tested by ELISA. The concentrations of each toxin were compared as unadjusted concentrations (ng/ml) and corrected for organism inoculum for each treatment (ng/ml to log_10_ colony-forming units, CFU/ml). Ebselen significantly suppressed toxin production in MRSA USA300 (Fig. 2).

### Activity against biofilms

**Figure 3:**
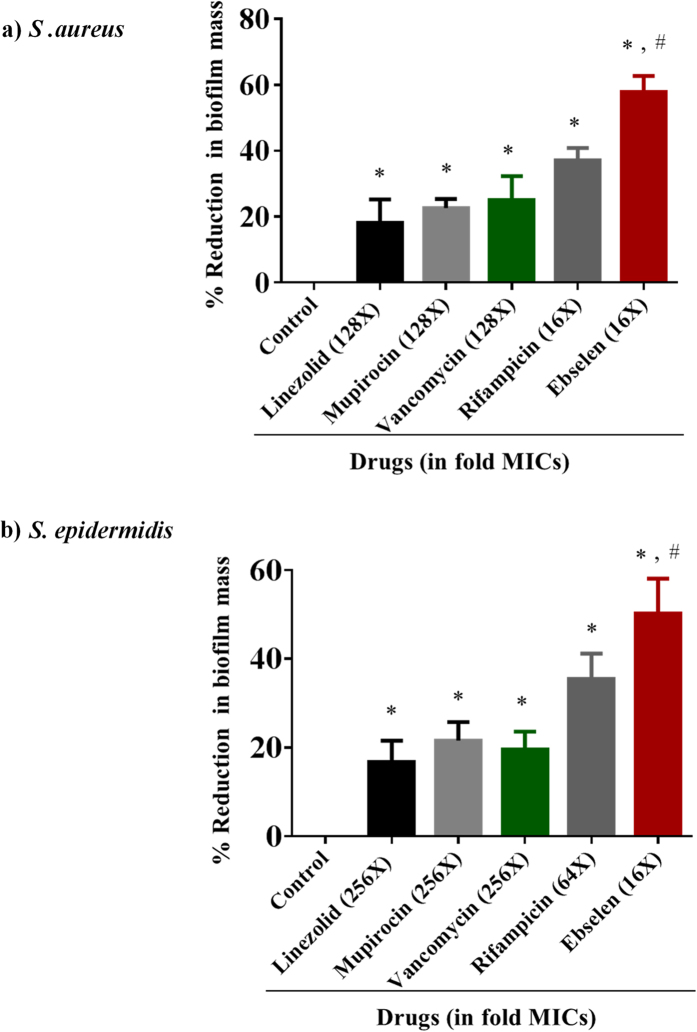
The effects of ebselen and antibiotics (linezolid, mupirocin, vancomycin and rifampicin) on established biofilms of *S. aureus* (a) or *S. epidermidis*(b). The established biofilms were treated with control antibiotics or ebselen and stained with crystal violet. Optical density of dissolved crystal violet was measured using a spectrophotometer. Values are the mean of triplicate samples with the standard deviation bars. *P* values of (*,#*P* ≤ 0.05) are considered as significant. Ebselen was compared to controls (*) and to antibiotics (#). Detailed *P* values are listed: *S. aureus* (**a**): Control vs linezolid, 0.0214; Control vs mupirocin, 0.0001; Control vs vancomycin, 0.004; Control vs rifampicin, 0.0001; Control vs ebselen, 0.0001; linezolid vs ebselen, 0.0014; mupirocin vs ebselen, 0.0004; vancomycin vs ebselen, 0.003; rifampicin vs ebselen, 0.0047. *S. epidermidis* (**b**): Control vs linezolid, 0.0036; Control vs mupirocin, 0.0008; Control vs vancomycin, 0.0011; Control vs rifampicin, 0.0004; Control vs ebselen, 0.0004; linezolid vs ebselen, 0.0033; mupirocin vs ebselen, 0.0052; vancomycin vs ebselen, 0.0040; rifampicin vs ebselen, n.s (not significant).

Considering the excellent broad-spectrum activity of ebselen against the MRSA and VRSA strains, we also considered the possibility that ebselen would be active against established biofilm. Biofilm-forming strains of *S. aureus* and *S. epidermidis* were used and the biofilm mass was estimated after treatment with ebselen and control antibiotics. Ebselen was significantly superior in reducing adherent biofilms of both *S. aureus* and *S. epidermidis* when compared to conventional antibiotics (linezolid, mupirocin, vancomycin and rifampicin). Ebselen at (2 μg/ml)(16X MIC) significantly reduced the biofilm mass, approximately by 60%. Control antibiotics, such as linezolid (256 μg/ml) (128X MIC), mupirocin (16 μg/ml) (128X MIC), and vancomycin (128 μg/ml) (128X MIC), were able to reduce the biofilm mass only by 20%. Rifampicin at 0.5 μg/ml (16X MIC) reduced the biofilm mass by only 40% (Fig. 3a).

Ebselen at 8 μg/ml (16X MIC), significantly reduced the strong biofilms of *S. epidermidis,* by more than 50%. However, linezolid (512 μg/ml) (256X MIC), mupirocin (32 μg/ml) (256X MIC), and vancomycin (256 μg/ml) (256X MIC) reduced biofilm mass by only 20% and rifampicin (2 μg/ml) (64X MIC) reduced biofilm mass by 40% (Fig. 3b).

### Cytotoxicity study

**Figure 4:**
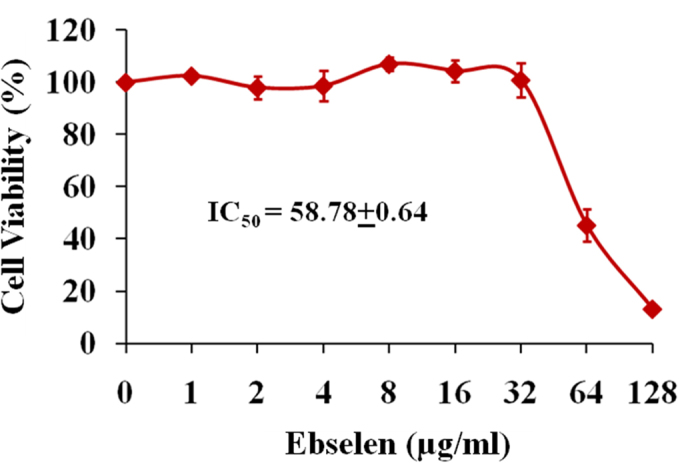
Cytotoxicity assay in human keratinocyte (HaCat) cells. HaCat cells were treated with different concentration of ebselen ranging from 0 to 128 μg/ml. DMSO was used as a negative control. Cell viability was measured by MTS assay and IC_50_ of ebselen to cause cytotoxicity in HaCat cells was calculated.

Safety of ebselen in mammalian cells was evaluated against human keratinocyte cells (HaCat) by MTS assay. Ebselen did not show toxicity up to 32 μg/ml. The results demonstrated that half maximal inhibitory concentration (IC_50_) required by ebselen to inhibit 50% of HaCat cells was found to be 58.78 + 0.64 μg/ml (Fig. 4).

### The therapeutic efficacy of ebselen in a mouse model of MRSA skin infection

**Figure 5:**
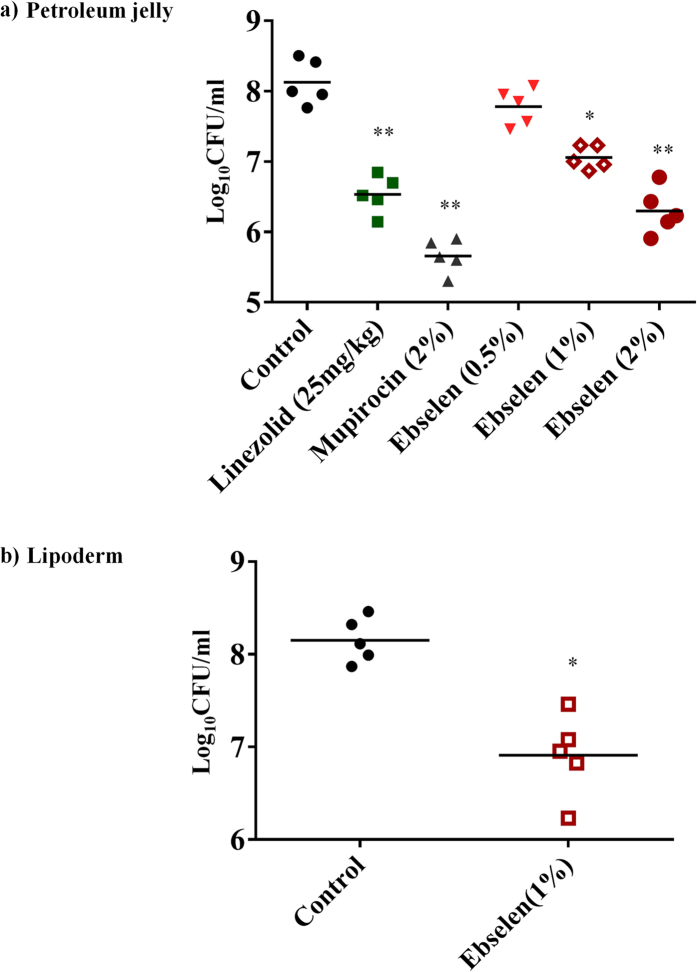
Efficacy of treatment of MRSA skin lesions with ebselen 0.5, 1, and 2%, linezolid (25 mg/kg), mupirocin (2%) and petroleum jelly (negative control) twice daily for 5 days (**a**). Treatment with ebselen 1% and lipoderm (negative control) twice daily for 5 days (**b**). Statistical analysis was calculated by the two-tailed Student *t* test. *P* values of (*P ≤0.05) (**P ≤0.01) are considered as significant. (#). Detailed *P* values are listed: (**a**): Control vs 2% linezolid, 0.0038; Control vs 2% mupirocin, 0.0020; Control vs 1% ebselen, 0.0337; Control vs 2% ebselen, 0.0032. (**b**): Control vs 1% ebselen, 0.0280.

- Bacterial load:
- Five groups of mice were treated topically either with vehicle alone (petroleum jelly) or control antibiotic (2% mupirocin) or ebselen (0.5%, 1%, or 2%) twice a day for five days. One group of mice was treated with linezolid orally. As shown in Fig. 5a, ebselen (1% and 2%) significantly reduced the mean bacterial counts compared with the control group (*P* ≤ 0.01). The group treated with 2% mupirocin had the highest reduction in CFU (2.28 ± 0.25 log_10_), followed by 2% ebselen (1.71 ± 0.11 log_10_), linezolid (25 mg/kg) (1.55 ± 0.01 log_10_), and 1% ebselen (1.02 ± 0.17 log_10_).
- Effect of vehicle:

In order to investigate the effect of the vehicle on the efficacy of ebselen in the treatment of MRSA skin infections, two groups of mice were treated topically either with vehicle alone (lipoderm base)^17^ or ebselen 1% formulated in lipoderm base twice a day for five days. Ebselen 1% significantly reduced the mean bacterial counts by 1.37 ± 0.20 log_10_ compared with the control group (*P* ≤ 0.01) (Fig. 5b). Although ebselen formulated in lipoderm base had higher reduction in bacterial count (1.37 ± 0.20 log_10_) than ebselen formulated in petroleum jelly (1.02 ± 0.17 log_10_), the difference was not statistically significant (Fig. 5a,b).

### Effect of ebselen on inflammatory cytokines induced by MRSA skin infection

**Figure 6:**
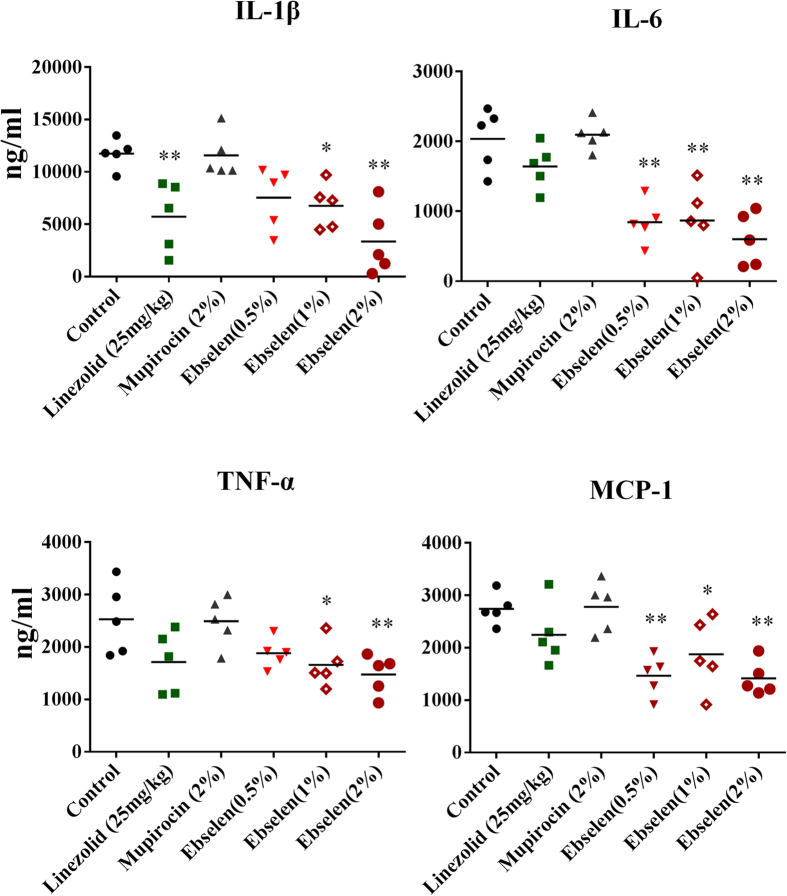
Effect of ebselen on cytokines production in MRSA skin lesions. Supernatants from skin homogenates were used for cytokine detection by ELISA. Each points represents single mice and each group has 5 mice. Statistical analysis was calculated by the two-tailed Student *t* test. *P* values of (*P ≤0.05) (**P ≤ 0.01) are considered as significant. Detailed *P* values are listed: IL-1β: Control vs 2% linezolid, 0.0054; Control vs 1% ebselen, 0.0026; Control vs 2% ebselen, 0.0007. IL-6: Control vs 0.5% ebselen, 0.0011; Control vs 1% ebselen, 0.0055; Control vs 2% ebselen, 0.0006. TNF-α: Control vs 1% ebselen, 0.0424; Control vs 2% ebselen, 0.0164. MCP-1: Control vs 0.5% ebselen, 0.0004; Control vs 1% ebselen, 0.0325; Control vs 2% ebselen, 0.0001.

To study the immune-modulatory activities of ebselen in a topical application against MRSA skin infection, we used ELISA to measure the pro-inflammatory cytokines tumor necrosis factor-α (TNF-α), interleukin-6 (IL-6), interleukin-1 beta (IL-1β) and monocyte chemo attractant protein-1(MCP-1) in the infected wounds. As shown in Fig. 6, ebselen 2% and 1% significantly reduced all tested pro-inflammatory cytokines, including IL-6, IL-1β, TNF-α, and MCP-1. However, ebselen at 0.5% significantly reduced IL-6 and MCP-1 only. Ebselen had considerably higher anti-inflammatory activity compared to antibiotics (linezolid and mupirocin).

### Synergistic activity of ebselen with topical antimicrobials *in vitro*

**Figure 7:**
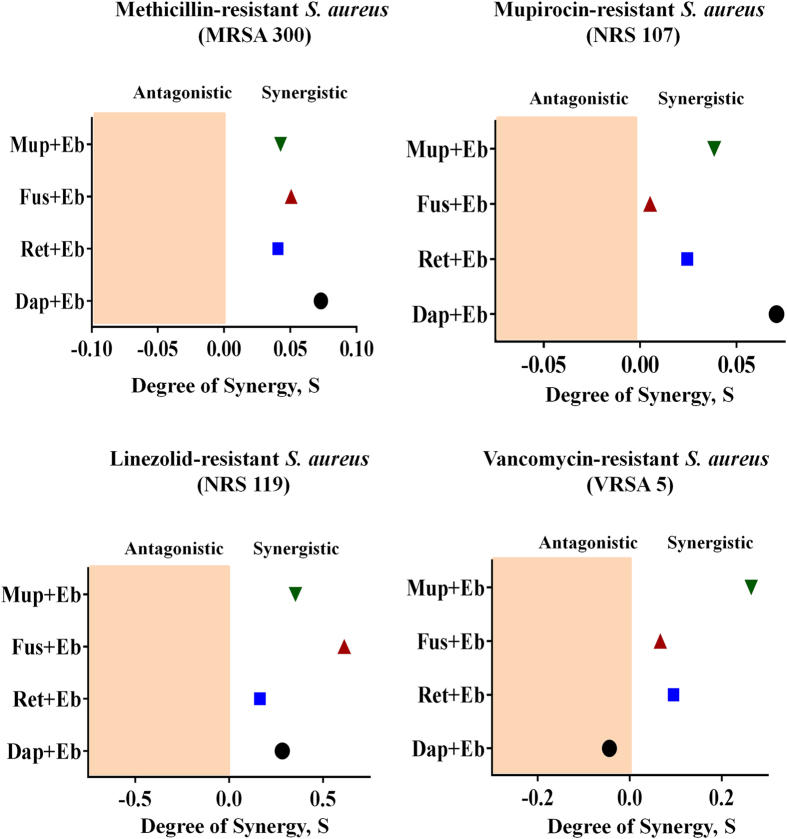
Synergistic activity of ebselen with topical antimicrobials. The Bliss Model for Synergy confirms a synergistic effect, between ebselen and topical antimicrobials (mupirocin, fusidic acid, retapamulin and daptomycin) against various resistant strains of *S. aureus*. Degree of synergy was quantified after 12 h of treatment with ebselen (0.0312 μg/ml) in combination with sub-inhibitory concentrations of topical antimicrobials. (Circle) daptomycin + ebselen, (Square) retapamulin + ebselen, (Triangle) fusidic acid + ebselen and (Inverted triangle) mupirocin + ebselen.

The antimicrobial activity of ebselen in combination with topical antimicrobials (mupirocin, fusidic acid, retapamulin and daptomycin) was investigated *in vitro* by the Bliss model of synergism against four clinical isolates. With the exception of the VRSA5 strain and the antibiotic daptomycin, ebselen acted synergistically with all tested antibiotics against *S. aureus* clinical isolates (Fig. 7).

## Discussion

For the past few decades the rise of multi-drug resistant *S. aureus* has been an emerging issue in hospital and community settings^5^,^18^. More importantly, the management of *S. aureus* strains associated with skin infections is becoming a serious issue in community settings^19^,^20^. Although there are several drugs recently approved by the FDA to combat Gram-positive pathogens such as tedizolid and dalbavancin^21^,^22^, there is still a pressing need for new antimicrobials to circumvent this burgeoning problem. Moreover, pharmaceutical companies are not interested in investing in antibiotic research and development because of low return compared to other drugs being developed for chronic ailments^5^,^23^,^24^. As an alternative to the traditional *de novo* antibiotic development, repurposing non-antimicrobial drugs is a novel and less expensive way to speed up the drug-development process^7^.

In an intensive search for antimicrobial activity among non-antibiotic drugs, we and others^10^,^15^ identified ebselen as a potent antimicrobial agent against Gram-positive pathogens including MRSA. Ebselen, an organoselenium compound, is known to be clinically safe with a well-known pharmacology profile and it is currently undergoing clinical trials for the prevention and treatment of various disorders such as cardiovascular disease, arthritis, stroke, atherosclerosis, and cancer^12^,^25^,^26^,^27^,^28^. Ebselen showed potent bactericidal activity against multiple clinical isolates of MRSA, including MRSA USA100, USA200, USA500, USA1000, and USA1100, which are resistant to various antimicrobials, including penicillin, fluoroquinolone, macrolides, and aminoglycosides. It also showed potent activity against multidrug-resistant clinical isolates of *S. aureus* strains, including a linezolid-resistant strain (NRS119), vancomycin-resistant strains (VRSA1-VRSA10), and a mupirocin-resistant strain (NRS107). Moreover, ebselen demonstrated excellent activity against MRSA USA300, a community-associated strain responsible for outbreaks of staphylococcal skin and soft-tissue infections (SSTI) in the United States^29^.

Although the antimicrobial activity of ebselen has been reported before^10^,^15^, its mechanism of action in *S. aureus* and its *in vivo* efficacy have never been explored. Ebselen, in our study, inhibited protein synthesis in *S. aureus.* Inhibition of protein synthesis at a concentration equivalent to the MIC demonstrates that, protein synthesis is likely primary antibacterial mechanism of action of ebselen. In addition, secondary effects on DNA, RNA, lipid synthesis and to a lesser extent on cell wall synthesis were also noticed at higher concentrations (8X MIC). It is possible that disruption of protein synthesis could lead to downstream inhibition of other pathways. This provides valuable insight into ebselen’s potential target in *S. aureus*. However, further work is needed to identify the cellular target of ebselen in *S. aureus*. For treatment of infections caused by toxin-producing pathogens such as *S. aureus,* inhibition of protein synthesis is an important consideration in the selection of antimicrobial agents^30^. Because antimicrobials that suppress translation in *S. aureus* markedly suppress the formation of toxins such as PVL and Hla, which will lead to better treatment outcomes^30^,^31^,^32^,^33^. In the light of our results, showing potent inhibition of bacterial protein synthesis, we tested the effect of ebselen on production of two important toxins in MRSA USA300 (Hla and PVL) by ELISA. Ebselen significantly suppressed toxin production after 1 hour incubation with MRSA. Inhibition of protein synthesis and the subsequent inhibition of toxin production are great advantages of ebselen as an antimicrobial agent.

Bacterial biofilms, which serve to protect the bacteria and hinder penetration of antibacterial drugs, contribute significantly to the treatment failure of Staphylococcus infections^34^. Given the potent antibacterial activity of ebselen against planktonic multidrug-resistant strains, we also considered the possibility that ebselen would be active against established bacterial biofilms of *S. aureus* and *S. epidermidis* (a leading cause of hospital-acquired implant-based infections)^35^. Ebselen was superior in reducing adherent biofilms of both *S. aureus* and *S. epidermidis* when compared to conventional antibiotics (linezolid, mupirocin, vancomycin and rifampicin).

In view of our results demonstrating the potent antimicrobial and antibiofilm activities of ebselen *in vitro* against MRSA, we moved forward with an *in vivo* experiment in a mouse model of MRSA skin infection. Ebselen 1% and 2% in petroleum jelly significantly reduced the mean bacterial counts compared with the control group (P ≤ 0.01). The lipoderm base enhanced the antimicrobial activity of ebselen but the reduction in bacterial load was not significant from petroleum jelly vehicle.

Since the clinical severity of *S. aureus* skin infections is driven by the excess host pro-inflammatory cytokines rather than by bacterial burden^36^,^37^, ebselen with its recognized immune-modulatory, anti-inflammatory, and antioxidant activities^11^,^38^ has great potential for treatment of for treatment of skin infections^37^,^39^. In this study, topical treatment with ebselen 1 and 2% significantly reduced IL-1β, IL-6, TNF-α and MCP-1 which might benefit the healing of infected wounds^40^,^41^,^42^,^43^,^44^. Linezolid also inhibits IL-1β which is in line with previous findings^37^,^45^. Prolonged inflammation especially due to inflammatory cytokines such as IL-6, TNF-α, and MCP-1, greatly delays healing in chronic wounds^39^. Ebselen significantly (*P* ≤ 0.01) inhibits all three cytokines (IL-6, TNF-α, and MCP-1), which should provide a favorable outcome in wound healing^39^.

With the increasing incidence of MRSA strains resistant to topical drugs of choice, such as mupirocin and fusidic acid, combination therapies are being explored^46^,^47^,^48^,^49^. To investigate whether ebselen has the potential to act synergistically with topical antimicrobials against multidrug-resistant strains, the Bliss independence model was utilized^50^. Ebselen acted synergistically with topical antimicrobials against resistant strains of *S. aureus,* thus providing a strong platform to combine ebselen with topical antimicrobials in treating staphylococcal skin infections and reducing the likelihood of strains developing resistance to monotherapy.

As a novel drug candidate for treating topical MRSA infections, ebselen has many advantageous qualities, including (i) potent antibacterial activity against clinical isolates of MRSA irrespective of the resistant patterns, (ii) suppression of bacterial protein synthesis and suppression of toxin production, (iii) potent anti-biofilm activity, (iv) suppression of excess pro-inflammatory cytokines, and (v) synergistic action with topical antimicrobials. These results lay the foundation for further analysis and development of ebselen as a potential treatment/prophylaxis for infected wounds and skin infections of public-health importance.

## Materials and Methods

### Bacterial strains and reagents

*Staphylococcus* strains used in this study are presented in Table 1. Mueller-Hinton broth (MHB) was purchased from Sigma-Aldrich. Trypticase soy broth (TSB), Trypticase soy agar (TSA), and Mannitol salt agar (MSA) were purchased from Becton, Dickinson (Cockeysville, MD). Ebselen was purchased from (Adipogen corp, San Diego), vancomycin hydrochloride (Gold Biotechnology), linezolid (Selleck Chemicals), mupirocin (applichem, NE), and chloramphenicol (Sigma-Aldrich)

### Antibacterial assays

MICs of drugs and antibiotics were evaluated by broth micro dilution method in MHB according to the Clinical and Laboratory Standards Institute (CLSI)^34^. The MIC was interpreted as the lowest concentration of the drug that completely inhibited the visible growth of bacteria after incubating plates for at least 16 hrs at 37 °C. Each drug was tested in triplicate in at least two independent experiments and the highest MIC value was reported.

### Macromolecular synthesis assay

Macromolecular synthesis assay was carried out in *S. aureus* strain ATCC 29213. Briefly, 100 μl of *S. aureus* grown in TSB at exponential phase (OD600 = 0.2 to 0.3), was added to triplicate wells and different concentrations of ebselen and control antibiotics (ciprofloxacin, rifampicin, linezolid, vancomycin and cerulenin) was added. DMSO treated cells served as a negative control. Cells treated with drugs and DMSO were incubated at 37 °C to allow the drug to act on bacterial cells. After 30 min incubation, radio labeled precursors for DNA, RNA, protein, cell wall and lipid synthesis such as [3H] thymidine (0 5μCi), [3H] uridine (0.5 μCi), [3H] leucine (1.0 μCi), [14C] N-acetylglucosamine (0.4 μCi), [3H] glycerol (0.5 μCi), respectively, were added for each reaction. After 15 min, reactions of DNA and RNA synthesis were stopped using 12 μl of 5% trichloroacetic acid (TCA). Similarly, protein synthesis was stopped after 40 min using 12 μl of 5% TCA. Reaction wells containing cell wall and lipid synthesis were stopped after 40 min using 100 μl of 8% SDS and 375 μl of chloroform/methanol (1:2) respectively. Reactions (DNA, RNA and protein) were incubated on ice for 30 min and the TCA precipitated materials were collected on a 25 mm GF/1.2 μM PES 96 well filter plate. After washing five times with cold 5% TCA, the filters were dried and counted using a Packard Top Count microplate scintillation counter. For cell wall synthesis, reaction tubes were then heated at 95 °C for 30 min, cooled, centrifuged, and spotted onto nitrocellulose membrane filters (0.8 μM). After washing three times with 0.1% SDS, the filters were rinsed two times with deionized water, allowed to dry, and then counted using a Beckman LS3801liquid scintillation counter. For lipid synthesis, reactions tubes were centrifuged at 13,000 rpm in a microfuge for 10 min, and then 150 μl of the organic phase was transferred to a scintillation vial and allowed to dry for at least 1 hour. Samples were then counted using liquid scintillation counting. Based on the incorporation of radiolabeled precursors of DNA, RNA, protein, cell wall and lipid synthesis, results were expressed as percent inhibition of macromolecular synthesis pathways.

### Measuring toxin production by ELISA

We tested the effect of ebselen on production of two important toxins Hla and PVL by ELISA as described before^30^,^51^. Briefly, Overnight grown MRSA USA300 bacterial culture was diluted approximately to 5 × 10^8^ CFU/ml in TSB. 10X MICs of drugs and antibiotics were added and incubated in the shaking incubator at 37 °C. After 1 hr the bacterial culture was centrifuged and the supernatants were used for toxin detection.

ELISA plates (Nunc) were coated with 2 μg/ml of sheep anti-Hla IgG (Toxin technology) in 100 μl of coating buffer and left overnight at 4 °C. Plates were then washed 3 times with Tris-buffered saline (TBS) containing 0.05% tween 20 (wash buffer) and then blocking solution containing TBS with 2% bovine serum albumin was added. After 1 hour incubation at 37 °C, plates were washed 3 times with wash buffer. A total of 100 μL of bacterial supernatants were added and incubated the plates at 37 °C for 2 hours. Purified Hla (Toxin technology) was used to generate a standard curve. Plates were again washed 3 times with wash buffer and 100 μL of sheep anti-Hla HRP conjugate at a dilution of 1:300 was added. After 1 hour of incubation at 37 °C and final washing, 100 μL of 3, 3′, 5, 5′-tetramethylbenzidine substrate (Sigma-Aldrich) was added, and the reaction was stopped after 10 minutes with 100 μL of 0.2N H2S04. Plates were read on a spectrophotometer at optical density (OD) 450, and data were analyzed with SoftMax Pro (Molecular Devices). The nominal range of this assay was 0.1–6 μg /mL.

For PVL Luk-S toxin, ELISA plates (Nunc) were coated as before with 2 μg /ml of mouse anti- PVL Luk-S monoclonal antibody (IBT Bioservices). Purified *S. aureus* LukS-PV (His-tag) (IBT Bioservices) was used to generate a standard curve. The experiment was carried as before except detection antibodies rabbit anti-PVL Luk-S (2 μg/ml) and rabbit IgG HRP conjugate (R&D Systems) at a dilution of 1:6000 was used. The concentrations of each toxin was compared as unadjusted concentrations (ng/ml) and corrected for organism inoculum for each treatment (ng/ml to log_10_ CFU/ml).

### Biofilm assay

Biofilm assay was performed as described before^34^. Briefly, biofilm-forming clinical isolates of *S. aureus* (ATCC 6538) and *S. epidermidis* (ATCC 35984) were inoculated in 96-well flat-bottom cell culture plates (polystyrene) in TSB supplemented with 1% glucose at 37 °C for 24 h. Then culture medium was removed, and wells were carefully washed with PBS four times to remove planktonic bacteria. Ebselen and antibiotics (linezolid, mupirocin, vancomycin and rifampicin) were added at different concentrations in TSB, and plates were incubated at 37 °C for 24 h. The wells were rinsed by submerging the entire plate in a tub containing tap water. Biofilms were stained with 0.1% (wt/vol) crystal violet for 30 min. After staining, the dye was removed and the wells were washed four times with water. The plates were dried for 1 h and ethanol (95%) was added to solubilize the dye bound to the biofilm. The OD of biofilm mass was measured at 595-nm absorbance by using a micro plate reader (Bio-Tek Instruments Inc.)

### Cytotoxicity assay

Human keratinocyte (HaCat) cells were seeded at a density of 10,000 cells per well in a 96-well tissue culture plate (CytoOne, CC7682-7596) in DMEM media containing 10% fetal bovine serum (FBS) and incubated overnight at 37 °C. Then cells were treated with ebselen at different concentrations from 0 to 128 μg/ml for 24 hours. Treated cells were washed four times with PBS and the DMEM media containing MTS assay reagent, 3-(4,5-dimethylthiazol-2-yl)-5-(3carboxymethoxyphenyl)-2-(4-sulfophenyl)-2H-tetrazolium) (Promega, Madison, WI, USA) was added. After 4 hrs of incubation at 37 °C, absorbance was measured using ELISA microplate reader (Molecular Devices, Sunnyvale, CA, USA). Percent cell viability of ebselen treated cells were calculated in relative to the untreated cells.

### Mice infection

All experiments were performed in accordance with relevant guidelines and regulations. Eight weeks old female BALB/c mice were used for this study (Harlan Laboratories, Indianapolis, IN). All animal procedures were approved by Purdue University Animal Care and Use Committee (PACUC). The murine model of MRSA skin infection has been described before^52^. Mice were injected intradermally with 40 μl of MRSA USA300 (1.65 × 10^8^) CFU per mouse. Forty-eight hours after infection and formation of open wound, the mice were divided into eight groups (n = 5). Four groups were treated topically with either 0.5%, 1%, or 2% ebselen in petroleum jelly (ointment- skin protectant) or 1% ebselen in lipoderm (dermal and transdermal delivery cream base). Two groups received the vehicles alone (petroleum jelly or lipoderm). One group was treated topically with 2% mupirocin in petroleum jelly and the last group was treated orally with linezolid (25 mg/kg). All groups were treated twice a day for 5 days. Twenty-four hours after the last treatment, the area around the wound was lightly swabbed with 70% ethanol and the wound was excised for bacterial counting on MSA after homogenization.

### Cytokines detection

Skin homogenates were centrifuged and the supernatants were used to detect the cytokine level by ELISA. Tumor necrosis factor-α (TNF-α), interleukin-6 (IL-6), interleukin-1 beta (IL-1β), and monocyte chemo attractant protein-1(MCP-1) Duo-set ELISA Kits (R&D Systems, Inc.) were used for the quantification of cytokines The experiment was carried out as per the manufacture instructions^53^.

### Bliss model of synergism

Synergism was calculated using the Bliss Independence Model, which calculates a degree of synergy using the formula: *S* *=* *(f*_*A0*_*/f*_*00*_*)(f*_*0B*_*/f*_*00*_*) − (f*_*AB*_*/f*_*00*_), where *f*_*AB*_ refers to bacterial growth rate in the presence of the combined drugs at a concentration *A*, for one of the antibiotics, and *B* for the ebselen; *f*_*A0*_ and *f*_*0B*_ refer to the bacterial growth rates in the presence of antibiotics (or) ebselen at a concentration of *A* and *B*, respectively; *f*_*00*_ refers to the bacterial growth rate in the absence of drugs; and *S* corresponds to the degree of synergy, a parameter that determines a synergistic interaction for positive values and an antagonistic interaction for negative ones. Growth rates at 12 hr are determined and the degree of synergism was calculated as described before^50^.

### Statistical analyses

Statistical analyses were assessed by Graph Pad Prism 6.0 (Graph Pad Software, La Jolla, CA). *P* values were calculated by the two-tailed Student *t* test. *P* values of <0.05 were considered as significant.

## Additional Information

**How to cite this article**: Thangamani, S. *et al.* Repurposing ebselen for treatment of multidrug-resistant staphylococcal infections. *Sci. Rep.* **5**, 11596; doi: 10.1038/srep11596 (2015).

## Figures and Tables

**Table 1:** Clinical isolates of *Staphylococcus*strains used in the study.

Strain type	Strain ID	eGenomics spa repeat motiff	Phenotypic properties	eGenomics spatype	Ridom spatype	Source
Methicillin-resistant *S. aureus* (MRSA)	USA100	TJMBMDMGMK	Resistant to ciprofloxacin, clindamycin, erythromycin	2	t002	United States (Ohio)
	USA200	WGKAKAOMQQQ	Resistant to clindamycin, methicillin erythromycin, gentamicin,	16	t018	United States (North Carolina)
	USA300	YHGFMBQBLO	Resistant to erythromycin, methicillin, tetracycline	2	t008	United States (Mississippi)
	USA400	–	Resistant to methicillin, tetracycline	–	–	United States (North Dakota)
	USA500	YHGCMBQBLO	Resistant to ciprofloxacin, clindamycin, erythromycin, gentamicin, methicillin, tetracycline, trimethoprim	7	t064	United States (Connecticut)
	USA700	UJGFMGGM	Resistant to erythromycin, methicillin	49	t126	United States (Louisiana)
	USA800	TJMBMDMGGMK	Resistant to methicillin	29	t088	United States (Washington)
	USA1000	ZDGDGDEB	Resistant to erythromycin, methicillin	–	t316	United States (Vermont)
	USA1100	–	Resistant to methicillin	–	–	United States (Alaska)
	ATCC 43300	–	Resistant to methicillin	–	–	United States (Kansas)
	ATCC BAA–44	–	Multidrug–resistant strain	–	–	Lisbon, Portugal
Linezolid-resistant *S. aureus*	NRS119 (Lin^r^)	YHGCMBQBLO	Resistant to linezolid	7	t064	United States (Massachusetts)
Mupirocin-resistant S. aureus	NRS 107	YHGGFMBQBLQ	Resistant to methicillin and mupirocin	59	t211	
Vancomycin-resistant *S. aureus* (VRSA)	VRS1	TJMGMK	Resistant to vancomycin	–	t062	United States
	VRS2	TJMBMDMGMK	Resistant to vancomycin, erythromycin, spectinomycin	–	t002	United States
	VRS3a	TJMBMDMGMK	Resistant to vancomycin	–	t002	United States
	VRS3b	–	Resistant to vancomycin	–	–	United States
	VRS4	TJMBMDMGMK	Resistant to vancomycin, erythromycin, spectinomycin	–	t002	United States
	VRS5	TJMBMDMGMK	Resistant to vancomycin	–	t002	United States
	VRS6	TJMGMK	Resistant to vancomycin	–	t062	United States
	VRS7	TJMBMDMGMK	Resistant to vancomycin, β-lactams	–	t002	United States
	VRS8	TJMBMDMGMK	Resistant to vancomycin	–	t002	United States
	VRS9	TJMBMDMGMK	Resistant to vancomycin	–	t002	United States
	VRS10	TJMBMDMGMK	Resistant to vancomycin	–	t002	United States
*S. epidermidis*	NRS101	–	Prototype biofilm producer; resistant to methicillin, gentamicin	–	–	United States
*S. aureus*	ATCC 6538	–	Prototype biofilm producing strain	–	–	–

**Table 2:** MICs of ebselen and antibiotics against clinical isolates of *Staphylococcus*strains.

Strain type	Strain ID	MICs (μg/ml)		
		Ebselen	Linezolid	Vancomycin
Methicillin resistant *S. aureus* (MRSA)	USA100	0.125	2	2
	USA200	0.125	2	1
	USA300	0.125	2	1
	USA400	0.5	2	1
	USA500	0.125	2	1
	USA700	0.125	4	1
	USA800	0.125	4	1
	USA1000	0.125	2	1
	USA1100	0.125	2	1
	ATCC 43300	0.125	2	1
	ATCC BAA-44	0.25	2	1
Linezolid-resistant *S. aureus*	NRS119	0.125	>16	1
Mupirocin-resistant *S. aureus*	NRS 107	0.125	2	1
Vancomycin-resistant *S. aureus* (VRSA)	VRS1	0.25	1	>16
	VRS2	0.25	1	8
	VRS3a	0.25	2	>16
	VRS3b	0.25	2	>16
	VRS4	0.125	2	>16
	VRS5	0.25	2	>16
	VRS6	0.25	2	>16
	VRS7	0.5	2	>16
	VRS8	0.125	2	>16
	VRS9	0.25	2	>16
	VRS10	0.25	2	>16
*S. epidermidis*	NRS101	0.5	2	2
*S. aureus*	ATCC 6538	0.125	2	1
